# Risk Assessment of Sarcopenia in Patients With Type 2 Diabetes Mellitus Using Data Mining Methods

**DOI:** 10.3389/fendo.2020.00123

**Published:** 2020-03-10

**Authors:** Mengzhao Cui, Xiaokun Gang, Fang Gao, Gang Wang, Xianchao Xiao, Zhuo Li, Xiongfei Li, Guang Ning, Guixia Wang

**Affiliations:** ^1^Department of Endocrinology and Metabolism, The First Hospital of Jilin University, Changchun, China; ^2^College of Computer Science and Technology, Jilin University, Changchun, China; ^3^Key Laboratory for Endocrine and Metabolic Diseases of Ministry of Health of China, Shanghai National Clinical Research Center for Endocrine and Metabolic Diseases, Shanghai Institute for Endocrine and Metabolic Diseases, Ruijin Hospital, Shanghai Jiaotong University School of Medicine, Shanghai, China

**Keywords:** sarcopenia, risk assessment, support vector machine, random forest, type 2 diabetes mellitus

## Abstract

**Purpose:** Sarcopenia is a geriatric syndrome, and it is closely related to the prevalence of type 2 diabetes mellitus (T2DM). Until now, the diagnosis of sarcopenia requires Dual Energy X-ray Absorptiometry (DXA) scanning. This study aims to make risk assessment of sarcopenia with support vector machine (SVM) and random forest (RF) when DXA is not available.

**Methods:** Firstly, we recruited 132 patients aged over 65 and diagnosed with T2DM in Changchun, China. Clinical data were collected for predicting sarcopenia. Secondly, we selected 3, 5, and 7 features out of over 40 features of patient's data with backward selection, respectively, to train SVM and RF classification models and regression models. Finally, to evaluate the performance of the models, we performed leave one out and 5-fold cross validation.

**Results:** When training the model with 5 features, the sensitivity, specificity, negative predictive value (NPV) and positive predictive value (PPV) were favorable, and it was better than the models trained with 3 features and 7 features. Area under the receiver operating characteristic (ROC) curve (AUC) were over 0.7, and the mean AUC of SVM models was higher than that of RF.

**Conclusions:** Using SVM and RF to make risk assessment of sarcopenia in the elderly is an option in clinical setting. Only 5 features are needed to input into the software to run the algorithm for a primary assessment. It cannot replace DXA to diagnose sarcopenia, but is a good tool to evaluate sarcopenia.

## Introduction

Sarcopenia is an age-related geriatric syndrome, it is characterized by loss of muscle mass, decrease of muscle strength and decline of physical performance. Quantitative and qualitative changes in skeletal muscle structure and function are involved in the aging-related loss of muscle function (1). It is related to falls, impaired cardio-respiratory function, metabolic diseases, disability and even death in the elderly (2). As aging population increases, sarcopenia is becoming more and more closely watched. Based on previous studies, patients with T2DM are subjected to a higher risk of sarcopenia due to diabetic complications and insulin resistance (3, 4). A study conducted in China has reported that participants with T2DM have 1.56 times higher risk of sarcopenia than healthy people (5). Therefore, patients with T2DM should concern more about sarcopenia.

The diagnosis of sarcopenia includes three parts, muscle mass, muscle strength and physical performance. The loss of muscle mass indicates an important role in sarcopenia, while its diagnosis requires CT, MRI, ultrasound, anthropometry, bioelectrical impedance analysis or DXA scan. Of these, DXA is widely recognized as the gold standard test. Though DXA is efficient, it has covered only a minority of medical institutions in the world. In some small cities or poor areas, the screening of sarcopenia is not available.

Sarcopenia has been reported to be associated with several factors, such as age, inadequate nutrients, low hormone levels, decreased physical activity and so on. During aging, inflammatory factors such as cytokines (e.g., tumor necrosis factor-alpha), advanced glycosylation products increased, aging appear to be related to a state of chronic low-grade inflammation (6), and the increase in inflammatory factors may impair blood flow by damaging the microvascular endothelium, and this impairment may exacerbate sarcopenia in the elderly (7). Furthermore, as aging, reduced testosterone and estrogen can cause a declining anabolic effect and increasing catabolic effect on muscle fibers (8–10). In the elderly, sufficient protein intake is another vital component to maintain and regain muscle mass (11, 12) and lack of protein and Vitamin D has been indicated as a risk factor of sarcopenia (13). The above indicators may help diagnose sarcopenia in clinical practice.

Machine learning (ML) is a computer-based data analysis method, and it is based on the assumption that a dataset may contain patterns that can identify outcomes (14). By iteratively learning from the data, ML makes computer detect the underlying patterns and create a model. Thus, ML models learn from examples rather than being programmed with rules, which is the main difference from traditional methods (15). The results obtained from ML can be more accurate and reliable. Recent studies have demonstrated that ML methods can provide better accuracy and discrimination to predict outcomes of surgical risk (16), pathological nodal metastasis for early oral squamous cell carcinoma (17), diagnosis of Parkinson's disease (18), and predict the treatment response and prognosis in acromegaly (19). Therefore, we presented the evaluation of sarcopenia with two data mining methods, using basic information and laboratory examinations of the patients to make risk assessment of sarcopenia to identify sarcopenia early in the elderly with T2DM. The methods we used are supervised learning algorithms: support vector machine(SVM) (20) and random forests(RF) (21). They learn the characteristics of a given dataset and generate a mathematical model from training data (22–24). Then we can infer a result with the training models from new data we haven't seen before.

## Methods

### Subjects

We recruited subjects from March 2017 to February 2018 in the department of endocrinology and metabolism of the First Hospital of Jilin University, and they all aged ≥ 65 and met the diagnostic criteria of T2DM proposed by WHO in 1999. The patients received at least half an hour of sunlight daily, winter extended appropriately and signed informed consent. Patients who had severe peripheral neuropathy, disuse muscle atrophy, malignant tumor, autoimmune disease, supplement of Vitamin D, severe cognitive disorder, took drugs affecting skeletal muscle metabolism in the last 3 months, remained long-term bedridden and failed to complete dual energy DXA, were excluded. Informed consent was obtained from all individual participants included in the study. The study complied with the Declaration of Helsinki and was approved by the ethics committee of the First Hospital of Jilin University.

### Clinical Data Collection

Basic information including gender, age, medications, lifestyles and medical history was investigated, and physical examinations such as height, weight and calf circumference were measured according to regular measurements. Besides, physical performance such as grip strength and 6-meter regular step speed were also inducted using a grip dynamometer (EH-101 electronic dynamometer, China) and a timer, respectively. Blood samples were collected to do some tests from venous blood of patients after an 8 h period of fasting. Of these, serum albumin level was determined by Bromocresol green method (7600-210 Hitachi automatic biochemical analyzer, Japan). 25-OH-Vitamin D was measured by liquid chromatography tandem mass spectrometry, and then serum was stored at −70°C until batch analysis for other analytes in a laboratory (Jilin Hehe medical examination co., LTD) Body composition of all subjects was measured by dual-energy X-ray absorptiometry (Luna Prodigy Advance, GE, America).

### Diagnosis of Sarcopenia

The sarcopenic subjects had a relative skeletal muscle mass index that passed the Asian Working Group for Sarcopenia (AWGS) cut-off of 7.0 kg/m^2^ for men and 5.4 kg/m^2^ for women. Since the loss of muscle mass dominates in the development of sarcopenia, we didn't take grip strength and step speed into account, so the patients with low muscle mass were diagnosed with sarcopenia in this study.

### Data Cleaning

Since our data mining methods demanded the dataset to be complete, but not all patients could take all the examinations. We made up the missing data with k-Nearest-Neighbor algorithm (kNN). In detail, For a feature with missing data, we first chose features with complete data which were also medically relative to the feature we wanted to clean, then we performed a kNN (with *k* = 10) on these features' data to find out the 10 nearest neighbors of the target patients, respectively, after that, we calculated the missing data of a patient with the mean value of its 10 nearest neighbors, and set it in the dataset as the data we would use in data mining methods.

### Statistical Analysis

In order to provide some evidence of choosing factors into the algorithm we conducted and to reduce computation complexity for feature selection, we first made statistical analyses using SPSS 24.0 software to find the differential factors between sarcopenia group and non-sarcopenia group. Continuous variables were summarized as means ± standard deviation or medians (25th, 75th percentiles) and categorical variables were denoted by counts and percentages. Characteristics of subjects and their blood levels of nutritional factors between two groups were compared using the chi-square test (when they are categorical data), Student's *t*-test (when they are continuous data that conform to normal distribution), and Mann–Whitney *U*-test (when they are continuous data that conform to abnormal distribution).

### Data Mining Methods

We implemented our algorithms with Python programming language and sk-learn scientific computing framework (25). We used SVM and RF to build models and make risk assessment of sarcopenia. Additionally, with these two data mining methods, we made risk assessment in two ways, respectively: classification and regression. By classification, we trained models to directly classify a patient's data to be either positive or negative. By regression, we trained models to predict the ASM/H^2^ from a patient's data, then combined with this person's gender information, to infer a positive or negative result based on AWGS consensus.

We collected more than 40 features including age, height, weight to find out which features are most effective in the algorithms. We also weighed the effort to get these features in medical diagnosis while selecting. We used a backward selection method, which randomly removes a feature, then compares the new result with the prior one, and leaves the feature out or adds it back accordingly.

### Evaluation of Data Mining Methods on Sarcopenia Assessment

To evaluate the performance of our models on assessment of sarcopenia, we performed k-fold cross validation, leave one out cross validation and calculated their sensitivity, specificity, negative predictive value (NPV) and positive predictive value (PPV). The receiver operating characteristic (ROC) curve and area under the ROC curve (AUC) were also conducted as another evaluation tool.

### K-Fold Cross Validation (26)

The k-fold cross validation could evaluate the performance of a classifier. It sorts the dataset randomly then partitions it into k independent folds with the same quantity of examples, after that, it takes the 1-fold out as a test set, and the remaining folds as a training set. With the test set and training set configured, SVM or RF is executed with this setting. The cross-validation process is repeated for k times. After k rounds, we shall gain k groups of middle results, then we can calculate a mean value with standard error rate with these middle results. The advantage of performing a k-fold cross validation is that, with a small dataset, we could acquire a relatively stable evaluation of our model, even if random sorting differentiated the result each time we execute our algorithms.

### Leave One Out Cross Validation

The leave one out cross validation is an extreme case of k-fold cross validation with minor modification. Instead of partitioning the whole dataset into k-folds, leave-one-out divides it into the number-of-subject folds, for example in our dataset, we had collected 132 patients' data, then we should first take the first person out as test set, the remaining 131 people as training set, executed SVM or RF, then we got the first person's prediction result; secondly we put the first person's data back, while taking the second person's data as test set, leaving the remaining 131 people's data as training set, then we got the second person's result. Finally, we should have 132 results with leaving each one out once as test set. We could then calculate the sensitivity, specificity, NPV and PPV from those results. The advantage of performing a leave one out cross validation is that, unlike k-fold cross validation, leave one out always acquires the same result whenever the algorithm is executed.

### ROC Curve and AUC

We conducted ROC curve with each k-fold cross validation, to evaluate the performance of our algorithms on the assessment of sarcopenia. ROC curve, together with AUC, is a method to illustrate how much better a binary classifier performs than a random guess. To plot an ROC curve, we first set several different threshold, such as 0.1, 0.2, 0.5, 0.7, 0.9, then we executed the data mining algorithm to produce a probability for each sample in the test set; we compared these probabilities with the threshold to generate several confusion matrix under each threshold; after that, we calculated the true positive rate (TPR) and false positive rate (FPR) from each confusion matrix. Finally, we plot the ROC curve and calculated the AUC. To be concise, we performed a similar numerical optimization method when calculating TPR and FPR, that is to first calculate the probabilities then set thresholds according to these probabilities, but it should be able to work out the same result as the method described first.

## Results

### Characteristics of Subjects

A total of 132 subjects were included in this study, and they were classified into the sarcopenia group and the non-sarcopenia group based on AWGS. As shown in Table 1, 38 cases had sarcopenia, accounting for 28.8%, with a median age of 73.5 years, and 94 cases had no sarcopenia, accounting for 71.2%, with a median age of 68. The sarcopenia group was significantly older than the non-sarcopenia group (*P* < 0.05). They also had a significantly lower appendicular skeletal muscle mass (ASM), ASM/H^2^, appendicular fat mass (AFM), trunk skeletal muscle mass (TSM), trunk fat mass (TFM), body mass index (BMI), grip strength and calf circumference than those of the non-sarcopenia group (*P* < 0.05), but there is little difference in gender, step speed, serum albumin, 25-OH-Vitamin D and 25-OH-Vitamin D3 between two groups.

**Table 1:** Baseline characteristics of subjects.

	**Sarcopenia (*n* = 38)**	**Non-sarcopenia (*n* = 94)**	***P***
Gender (male/female)	21 (55.3)/17 (44.7)	38 (40.4)/56 (59.6)	0.121
Age (years)	73.5 (68,77.25)	68 (65,72)	0.000^*^
Duration of diabetes (years)	14.0 (5.0,22.0)	13.0 (8.0,18.0)	0.590
History of hypertension	22 (57.9)	54 (57.4)	0.962
Smoking	10 (26.3)	24 (25.5)	0.926
Drinking	6 (15.8)	12 (12.8)	0.647
Exercise	20 (52.6)	65 (69.1)	0.073
High-protein diet	11 (28.9)	37 (39.4)	0.260
History of fall	12 (31.6)	18 (19.1)	0.123
ASM (kg)	16.42 (13.06,19.26)	17.65 (15.76,22.58)	0.001^*^
ASM/H2 (kg/m2)	5.67 (5.02,6.63)	6.90 (6.18,7.71)	0.000^*^
AFM (kg)	6.21 (4.45,7.70)	7.35 (5.44,8.66)	0.013^*^
TSM (kg)	20.99 ± 3.17	23.46 ± 3.86	0.001^*^
TFM (kg)	11.56 ± 4.27	14.55 ± 4.69	0.001^*^
BMI (kg/m2)	22.86 ± 2.71	26.34 ± 3.35	0.000^*^
Grip strength (kg)	19.65 (15.33,25.20)	22.15 (18.18,32.03)	0.013^*^
Step speed (m/s)	0.83 (0.71,0.93)	0.86 (0.81,0.92)	0.185
Calf circumference (cm)	33.3 (32.0,34.5)	35.5 (34.0,36.5)	0.000^*^
Serum albumin (g/L)	38.15 (34.53,42.23)	39.80 (37.88,42.40)	0.078
25-OH-Vitamin D (ng/mL)	19.43 ± 10.38	18.79 ± 7.22	0.686
25-OH-Vitamin D3 (ng/mL)	18.01 ± 10.32	16.43 ± 7.19	0.319

*ASM, appendicular skeletal muscle mass; AFM, appendicular fat mass; TSM, trunk skeletal muscle mass; TFM, trunk fat mass*.

* *Significantly different*.

*38 cases had sarcopenia, accounting for 28.8%, with a median age of 73.5 years, and 94 cases had no sarcopenia, accounting for 71.2%, with a median age of 68 years. The sarcopenia group was significantly older than the non-sarcopenia group (P < 0.05). They also had a significantly lower ASM, ASM/H^2^, AFM, TSM, TFM, BMI, grip strength and calf circumference than those of the non-sarcopenia group (P < 0.05), but there is little difference in gender, duration of diabetes, history of hypertension, smoking, drinking, exercise, high-protein diet, history of fall, step speed, serum albumin, 25-OH-Vitamin D and 25-OH-Vitamin D3 between two groups*.

### Sensitivity, Specificity, NPV, and PPV by SVM

As shown in Table 2, when we input 3 features: age, gender, and BMI, using leave one out of SVM, the sensitivity was 0.459–0.514, specificity was 0.947, NPV was 0.818–0.833 and PPV was 0.773–0.792. Then we input 5 features: age, gender, BMI, grip strength, calf circumference, the sensitivity, NPV and PPV were better than before. Additionally, 7 features: age, gender, BMI, grip strength, calf circumference, serum albumin, 25-OH-Vitamin D3 were used in the model, we found that there was little difference compared to 5 feature model but better than 3 feature model.

**Table 2:** Performance of SVM.

**Methods**	**Sensitivity**	**Specificity**	**NPV**	**PPV**
3 Features SVM classification^a^	0.459	0.947	0.818	0.773
3 Features SVM regression^a^	0.514	0.947	0.833	0.792
5 Features SVM classification^a^	0.541	0.947	0.841	0.8
5 Features SVM regression^a^	0.595	0.947	0.857	0.815
7 Features SVM classification^a^	0.568	0.947	0.848	0.808
7 Features SVM regression^a^	0.568	0.937	0.848	0.778
3 Features SVM classification^b^	0.523 ± 0.332	0.958 ± 0.069	0.829 ± 0.115	0.825 ± 0.244
3 Features SVM regression^b^	0.494 ± 0.318	0.948 ± 0.074	0.818 ± 0.111	0.781 ± 0.312
5 Features SVM classification^b^	0.530 ± 0.299	0.944 ± 0.072	0.849 ± 0.062	0.700 ± 0.415
5 Features SVM regression^b^	0.552 ± 0.315	0.944 ± 0.072	0.858 ± 0.065	0.700 ± 0.415
7 Features SVM classification^b^	0.425 ± 0.244	0.931 ± 0.070	0.818 ± 0.058	0.652 ± 0.388
7 Features SVM regression^b^	0.525 ± 0.335	0.945 ± 0.040	0.859 ± 0.072	0.692 ± 0.397

*3 features: age, gender, BMI*.

*5 features: age, gender, BMI, grip strength, calf circumference*.

*7 features: age, gender, BMI, grip strength, calf circumference, serum albumin, 25-OH-Vitamin D3*.

a *SVM by leave one out*.

b *SVM by 5-fold*.

*This table shows the results from our SVM algorithm. While operating SVM on our dataset, we set kernel to be linear, C = 1 or 2 in model training. We scaled our datasets with MinMaxScaler, which transforms the training set to be between (0, 1). Then performed the same scaler on test sets. The best classifier is 5 features SVM regression method, which is slightly better than 7 feature methods*.

When we performed by 5-fold of SVM, interestingly, we could see the sensitivity and NPV of 5 features were the best compared to 3 feature model and 7 feature model, and specificity and PPV were also higher than 0.8 (Table 2).

### Sensitivity, Specificity, NPV, and PPV by RF

Alternatively, we performed RF, as listed in Table 3, when using 5 features, the sensitivity and NPV were better than the other two groups. Integrally, the results of 7 feature model were better than 3 feature model and similar to 5 feature model. Moreover, we conducted 5-fold of RF, the sensitivity, NPV, and PPV of 5 feature model were apparently better than the other two. Taken together, the results of 5 and 7 feature model were better than 3 feature model.

**Table 3:** Performance of RF.

**Methods**	**Sensitivity**	**Specificity**	**NPV**	**PPV**
3 Features RF classification^a^	0.459	0.895	0.81	0.63
3 Features RF regression^a^	0.405	0.937	0.802	0.714
5 Features RF classification^a^	0.459	0.895	0.81	0.63
5 Features RF regression^a^	0.486	0.895	0.817	0.643
7 Features RF classification^a^	0.432	0.926	0.807	0.696
7 Features RF regression^a^	0.432	0.916	0.806	0.667
3 Features RF classification^b^	0.492 ± 0.193	0.909 ± 0.063	0.815 ± 0.100	0.703 ± 0.212
3 Features RF regression^b^	0.373 ± 0.155	0.941 ± 0.078	0.789 ± 0.083	0.752 ± 0.246
5 Features RF classification^b^	0.546 ± 0.172	0.938 ± 0.066	0.848 ± 0.045	0.771 ± 0.255
5 Features RF regression^b^	0.521 ± 0.167	0.894 ± 0.064	0.834 ± 0.041	0.663 ± 0.119
7 Features RF classification^b^	0.464 ± 0.171	0.934 ± 0.066	0.824 ± 0.045	0.777 ± 0.137
7 Features RF regression^b^	0.448 ± 0.199	0.901 ± 0.079	0.818 ± 0.024	0.640 ± 0.152

*3 features: age, gender, BMI*.

*5 features: age, gender, BMI, grip strength, calf circumference*.

*7 features: age, gender, BMI, grip strength, calf circumference, serum albumin, 25-OH-Vitamin D3*.

a *RF by leave one out*.

b *RF by 5-fold*.

*This table shows the result of random forests algorithm. We didn't perform data scaling while performing RF. We set number of estimators to be 100 and max depth to be 5 in the hyper parameters of random forests classifier and regressor. The sensitivity of 5 features models are better than others, the specificity of 7 features models are the best among six models*.

### ROC Curve by SVM and RF

**Figure 1:**
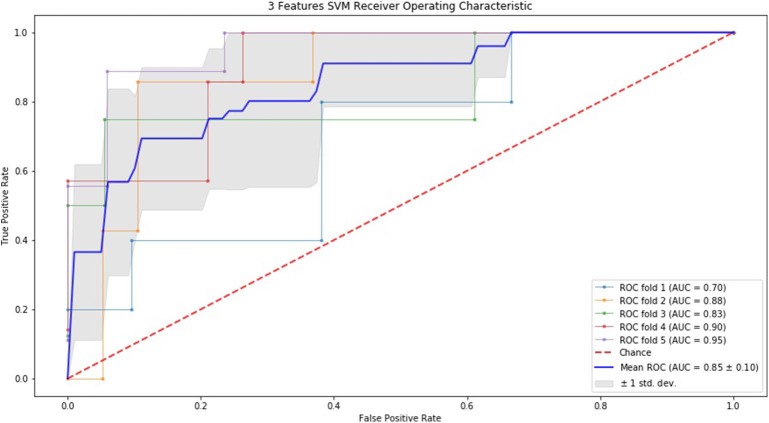
ROC of 5-fold cross validation results of SVM. The mean AUC of 3 feature model was 0.85 ± 0.10.

**Figure 2:**
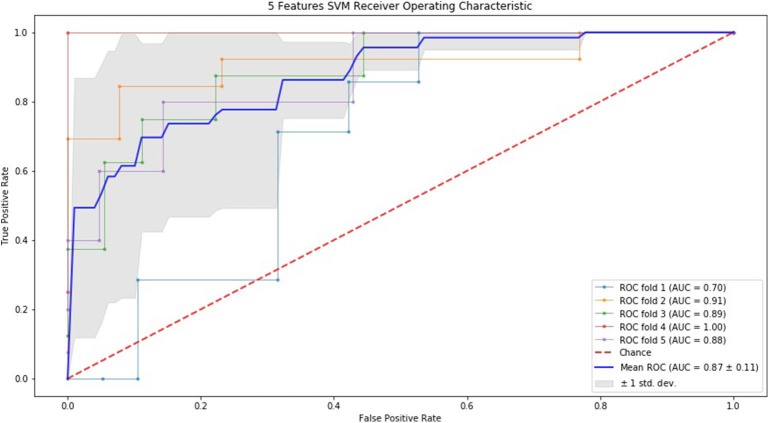
ROC of 5-fold cross validation results of SVM. The mean AUC of 5 feature model was 0.87 ± 0.11.

**Figure 3:**
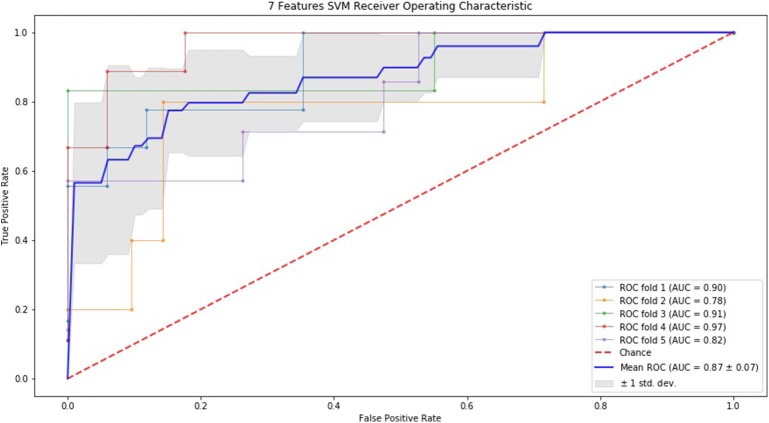
ROC of 5-fold cross validation results of SVM. The mean AUC of 7 feature model was 0.87 ± 0.07.

**Figure 4:**
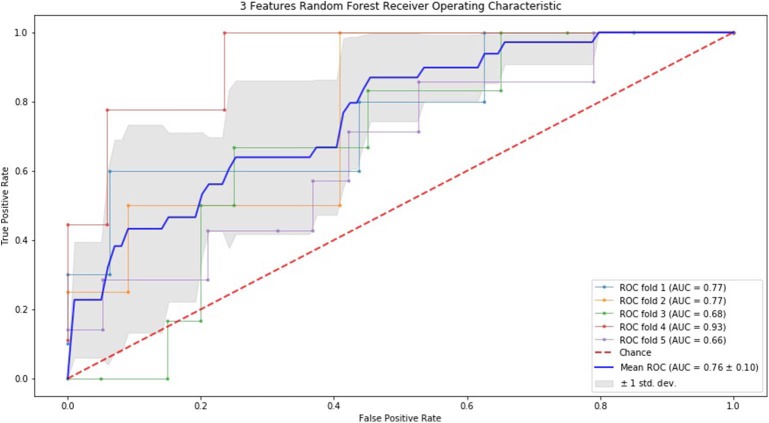
ROC of 5-fold cross validation results of RF. The mean AUC of 3 features was 0.76 ± 0.10.

**Figure 5:**
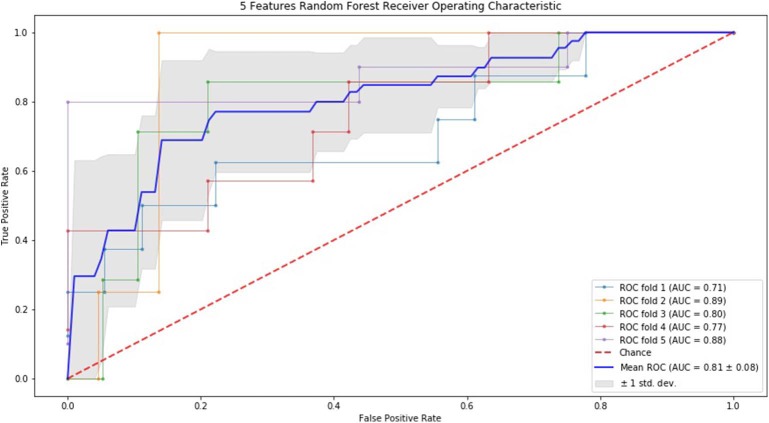
ROC of 5-fold cross validation results of RF. The mean AUC of 5 features was 0.81 ± 0.08.

**Figure 6:**
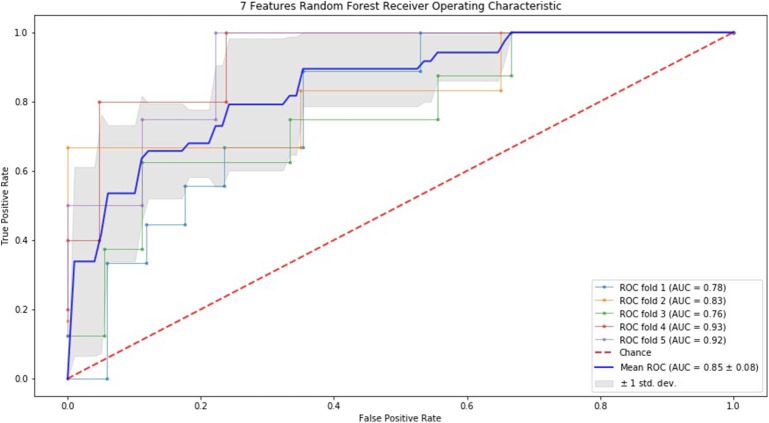
ROC of 5-fold cross validation results of RF. The mean AUC of 7 features was 0.85 ± 0.08.

Finally, we performed ROC curve and AUC using 5-fold of SVM (Figures 1–3) and RF (Figures 4–6), respectively. First, with SVM, mean AUC of 5 feature model and 7 feature model were similar (0.87 ± 0.11, 0.87 ± 0.07, respectively), and they were higher than that of 3 feature model (0.85 ± 0.10). Then, with RF, the mean AUC of 3 features was 0.76 ± 0.10, of 5 features was 0.81 ± 0.08, and highest of all was 0.85 ± 0.08 with 7 features.

## Discussion

To date, this is the first study that aims at making risk assessment of sarcopenia in the elderly with T2DM using data mining methods. The evaluation of loss of muscle mass requires DXA scan, which is known as the gold standard, but in some small medical institutions or some poor areas, this examination is not available. For this purpose, we put forward data mining methods, using as few factors as possible to make risk assessment of sarcopenia in the elderly with T2DM preliminarily. If an elderly is at a high risk of sarcopenia based on the result of our methods, then we suggest this person should focus on sarcopenia and may perform a DXA further to acquire a definitive diagnosis.

As to the indicators selected in this study, according to the backward selection, we selected factors like age, gender and BMI as the favorable ones to build models, in addition, we also took clinical experience into account, as well as the studies reported all over the world. Nutrient status are deemed to related factors already, and 25-OH-Vitamin D3 is the major form in circulation, it's a good indicator of vitamin D status. Therefore, we chose serum albumin and 25-OH-Vitamin D3 as the assessment of nutrients of these patients to build model using data mining methods. In addition, Table 1 shows that sarcopenia group had a significantly lower BMI, calf circumference and grip strength than non-sarcopenia group. The loss of muscle mass in patients with sarcopenia may lead to the low metabolic level of the body, so the patients could be characterized by low BMI and calf circumference. Besides, decline of muscle strength is also a feature of sarcopenia, so grip strength is thought to be a suitable factor to assess sarcopenia.

This study revealed the sensitivity, specificity, NPV and PPV of SVM and RF using both leave one out method and 5-fold method, respectively. From Table 2 to Table 3, we suggested that when using 5 features to build the model, the above results were almost acceptable, that means it was better than the result of using 3 features and not worse than that of using 7 features even better than it. Although the sensitivity was all in a low level with each method, the specificity, NPV and PPV were in a higher level. In addition, we performed ROC curve and AUC to verify the value of both algorithms. All AUCs were over 0.7, indicating a favorable value of each method. For SVM, the mean AUC was a little higher than that of RF, we believe SVM is more reliable than RF in terms of our models. Apart from SVM and RF, we also built a model based on artificial neural network (27, 28) (also known as machine learning), but this model tended to be overfitting no matter how we adjusted the parameters, we believe the main reason is that our dataset was not big enough to fit a two to three layer neural network. On our 132-sample dataset, the performance of our algorithm on training set and test set were similar, they tended not to be underfitting or overfitting with proper parameters. So, we believe SVM and RF are more applicable than artificial neural networks on risk assessment of sarcopenia with small datasets. Undoubtedly, DXA is the gold-standard diagnosis of muscle mass, but our results indicate a possibility of using these two algorithms to make primary risk assessment of sarcopenia, in this sense, it can be considered to be used to assess a person necessarily when DXA is not available.

As for limitations of this study, the small number of samples especially lack of patients with definitive diagnosis of sarcopenia is the major point and the problem of unbalanced population exists indeed. However, sarcopenia is a chronic disease, its short-term effects on the health are not apparent. In this regard, our low sensitivity may be accepted for those people who have no access to DXA. In the future, we should collect more samples to improve the precision of the algorithm. Meanwhile, apart from the features that are continuous values, such as age, glycated hemoglobin and others, there are features that are binary, for example, did a patient smoke, was a patient an office worker or a farmer, has a patient been taking a specific medicine for a long time? These discrete values were taken into modeling, but because they are mostly skewed, the performance of our model wasn't better. For example, not a lot of patients have a history of previous falls, even if hypothetically a previous fall history is a significant factor for the model, due to the skewed data we got, it didn't increase the performance of our models. Although we left out a lot of features, and only formed our models with 3, 5, or 7 features, the features left out could still contribute to potential studies on diagnosing sarcopenia if data could be properly collected.

In conclusion, using data mining methods including SVM and RF to make risk assessment of sarcopenia in the elderly is an option in clinical setting. Age, gender, BMI, grip strength and calf circumference are only needed to input into the algorithm to make a primary assessment. Although it's far from the gold standard to diagnose sarcopenia, in some cases, it's still a good tool to evaluate sarcopenia.

## Data Availability Statement

The datasets generated for this study are available on request to the corresponding author.

## Ethics Statement

The studies involving human participants were reviewed and approved by Ethics Committee of the First Hospital of Jilin University. The participants provided their written informed consent to participate in this study.

## Author Contributions

XG, GuW, and GN contributed conception and design of the study. XL organized the database. XX and GaW performed the statistical analysis. MC and FG wrote the first draft of the manuscript. ZL wrote sections of the manuscript. All authors contributed to manuscript revision, read, and approved the submitted version.

### Conflict of Interest

The authors declare that the research was conducted in the absence of any commercial or financial relationships that could be construed as a potential conflict of interest.

## Abbreviations

AFM: appendicular fat mass
ASM: appendicular skeletal muscle mass
AUC: area under the receiver operating characteristic curve
AWGS: Asian Working Group for Sarcopenia
BMI: body mass index
DXA: dual energy X-ray absorptiometry
FPR: false positive rate
H: eight
IL-1: interleukin-1
IL-6: interleukin-6
kNN: k-Nearest-Neighbor algorithm
ML: machine learning
NPV: negative predictive value
PPV: positive predictive value
RF: random forest
ROC: receiver operating characteristic
SVM: support vector machine
T2DM: type 2 diabetes mellitus
TFM: trunk fat mass
TNF-α: tumor necrosis factor-α
TPR: true positive rate
TSM: trunk skeletal muscle mass.
